# Adapting the Comprehensive Unit Safety Program (CUSP) implementation strategy to increase delivery of evidence-based cardiovascular risk factor care in community mental health organizations: protocol for a pilot study

**DOI:** 10.1186/s43058-021-00129-6

**Published:** 2021-03-04

**Authors:** Emma Elizabeth McGinty, David Thompson, Karly A. Murphy, Elizabeth A. Stuart, Nae-Yuh Wang, Arlene Dalcin, Elizabeth Mace, Joseph V. Gennusa, Gail L. Daumit

**Affiliations:** 1 Johns Hopkins University Bloomberg School of Public Health, 624 N. Broadway, Room 359, Baltimore, MD 21205 USA; 2 Johns Hopkins University School of Medicine, 733 N. Broadway, Baltimore, MD 21202 USA

**Keywords:** Serious mental illness, Integrated care, Cardiovascular, Culture, Self-efficacy

## Abstract

**Background:**

People with serious mental illnesses (SMI) such as schizophrenia and bipolar disorder experience excess mortality driven in large part by high rates of poorly controlled and under-treated cardiovascular risk factors. In the USA, integrated “behavioral health home” models in which specialty mental health organizations coordinate and manage physical health care for people with SMI are designed to improve guideline-concordant cardiovascular care for this group. Such models have been shown to improve cardiovascular care for clients with SMI in randomized clinical trials, but real-world implementation has fallen short. Key implementation barriers include lack of alignment of specialty mental health program culture and physical health care coordination and management for clients with SMI and lack of structured protocols for conducting effective physical health care coordination and management in the specialty mental health program context. This protocol describes a pilot study of an implementation intervention designed to overcome these barriers.

**Methods:**

This pilot study uses a single-group, pre/post-study design to examine the effects of an adapted Comprehensive Unit Safety Program (CUSP) implementation strategy designed to support behavioral health home programs in conducting effective cardiovascular care coordination and management for clients with SMI. The CUSP strategy, which was originally designed to improve inpatient safety, includes provider training, expert facilitation, and implementation of a five-step quality improvement process. We will examine the acceptability, appropriateness, and feasibility of the implementation strategy and how this strategy influences mental health organization culture; specialty mental health providers’ self-efficacy to conduct evidence-based cardiovascular care coordination and management; and receipt of guideline-concordant care for hypertension, dyslipidemia, and diabetes mellitus among people with SMI.

**Discussion:**

While we apply CUSP to the implementation of evidence-based hypertension, dyslipidemia, and diabetes care, this implementation strategy could be used in the future to support the delivery of other types of evidence-based care, such as smoking cessation treatment, in behavioral health home programs. CUSP is designed to be fully integrated into organizations, sustained indefinitely, and used to continually improve evidence-based practice delivery.

**Trial registration:**

ClinicalTrials.gov, NCT04696653. Registered on January 6, 2021

Contributions to the literature

- This pilot study examines an implementation strategy designed to overcome key barriers to integrating physical health care coordination and management in specialty mental health settings.
- This protocol describes a pilot study examining whether an adapted Comprehensive Unit Safety Program (CUSP) implementation strategy, designed for inpatient safety, can support the implementation of integrated physical health care in specialty mental health organizations.
- The study described in this protocol is the first to develop and implement an evidence-based practice “bundle” integrating guideline-concordant clinical and cross-cutting cardiovascular risk factor care processes for people with serious mental illness.

## Introduction

### Cardiovascular risk among people with SMI

People with serious mental illnesses (SMIs) such as schizophrenia and bipolar disorder experience 10–20 years of premature mortality relative to the overall population [1–4]. This excess mortality is driven by high rates of poorly controlled cardiovascular disease and other somatic conditions [2, 5, 6]. Prevalence of cardiovascular disease and its risk factors, including hypertension, dyslipidemia, and diabetes mellitus, is elevated among people with SMI for multiple interrelated reasons, including metabolic side effects of the psychotropic medications many people with SMI need to use to control their psychiatric symptoms and high rates of social risk factors for cardiovascular disease, including poverty, unemployment, and criminal justice involvement [7–13].

### Barriers to guideline-concordant cardiovascular care for people with SMI

In the USA, low rates of guideline-concordant care contribute to poor control of cardiovascular disease and its risk factors among individuals with SMI, particularly those insured by Medicaid [14, 15]. The historic separation of the USA specialty mental health system from the general medical system impedes the delivery of guideline-concordant cardiovascular care for people with SMI due to challenges in sharing information and coordinating care across systems [16–18]. Medical training is also fragmented; specialty mental health providers lack training and experience in managing cardiovascular risk, and general internists lack training and experience in managing serious mental illness [19, 20]. Further, the lack of social support and cognitive and communication impairments experienced by some people with SMI can make navigating care across complex health systems very challenging [11, 21–23].

## Integrated care models

Over the past decade, integrated care delivery models designed to overcome barriers to care driven by general medical-specialty mental health system fragmentation have become increasingly common in the USA. Prominent examples include primary care-based models, including Collaborative Care and Primary Care Medical Homes (PCMHs) [24–26], as well as specialty mental health-based models [27, 28], which are the focus of the study described in this protocol. People with SMI receive much of their care in the specialty mental health system and often have stronger relationships with their mental health providers than with their primary care providers, making specialty mental health system-based models an appealing setting for leading efforts to improve mental-physical care coordination for this group. Integrated care models based in the specialty mental health systems are often termed “behavioral health home” programs, or programs where specialty mental health providers are responsible for physical health care coordination and management for clients with SMI [27, 28]. Evidence from randomized clinical trials suggests that this type of specialty mental health organization-based integration model can increase guideline-concordant care for cardiovascular risk factors and other somatic conditions [29–31].

Historically, the lack of insurance reimbursement mechanisms for physical-mental health care coordination has been a major barrier to the implementation of integrated care models [32, 33]. However, recent innovations have made progress in overcoming this barrier, most notably the Centers for Medicare and Medicaid Services (CMS) behavioral health integration billing codes, which allow primary care providers to bill for mental health care coordination and management services [34], and the Affordable Care Act Medicaid Health Home waiver, which allows state Medicaid programs to create “health home” programs for subgroups of high-need, high-cost beneficiaries including but not limited to those with SMI [35]. This waiver allows states to bill Medicaid for care coordination and management services delivered to health home participants. To date, 17 states and Washington, D.C. have used the waiver to create behavioral health home programs [36].

### Need for scalable implementation interventions to support integrated care models

While financing mechanisms are a necessary component of care integration, reimbursement schemes have proven insufficient, on their own, to improve cardiovascular care quality and health outcomes among people with SMI. “Real-world” behavioral health homes have been associated with improved rates of screening and monitoring of cardiovascular conditions, reductions in somatic acute care use, and improved post-hospitalization follow-up [27]. However, behavioral health home programs implemented outside the clinical trial context have not yet improved the quality of cardiovascular care or cardiovascular health outcomes among people with SMI [27, 37, 38]. The lack of alignment of specialty mental health program culture with physical health care coordination and management for clients with SMI and the lack of structured protocols for conducting effective physical health care coordination and management in the specialty mental health program context have been identified as major behavioral health home implementation barriers [27, 39–42]. This protocol describes a pilot study of an implementation intervention designed to overcome these barriers.

### Study aims

In a pilot study guided by the Translating Evidence into Practice (TRIP) framework [43], we will test an adapted Comprehensive Unit Safety Program (CUSP) implementation strategy designed to support behavioral health home programs in conducting effective cardiovascular care coordination and management for clients with SMI. Using a single-group, pre/post-study design, we will examine the acceptability, appropriateness, and feasibility of the implementation strategy and how this strategy influences mental health organization culture; specialty mental health providers’ self-efficacy to conduct evidence-based cardiovascular care coordination and management; and receipt of guideline-concordant care for hypertension, dyslipidemia, and diabetes mellitus among people with SMI. We hypothesize that the CUSP implementation strategy will improve the two primary outcomes of organization quality improvement culture and provider self-efficacy. These outcomes are the mechanisms by which CUSP has been shown, in other contexts, to improve care quality [44–46]. This study was approved by the Johns Hopkins University School of Medicine Institutional Review Board (IRB00269855)

## Methods

### Conceptual framework

**Fig. 1:**
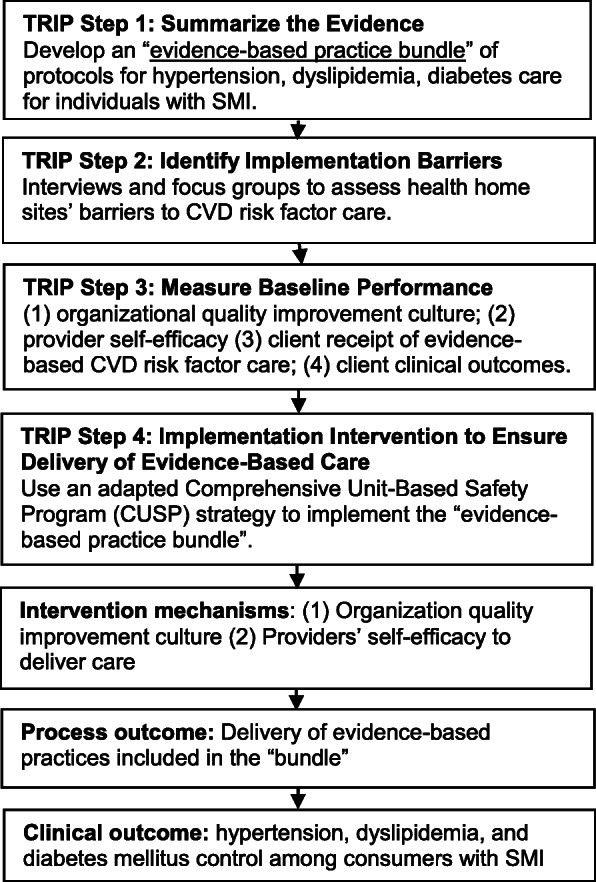
Conceptual framework: Translating Research into Practice (TRIP)

This pilot study is guided by the Translating Evidence into Practice (TRIP) model’s four steps for large-scale knowledge translation (Fig. 1) [43]. In step 1, summarize the evidence, the goal is to identify the specific evidence-based practices that will be implemented. Identification of specific practices is based on both strengths of evidence and feasibility of implementation [47, 48]. These practices are then delineated in a “bundle” of protocols with detailed information regarding, in this case, the conduct of clinical-guideline concordant hypertension, dyslipidemia, and diabetes mellitus care for people with SMI. Step 2 is focused on identifying local implementation barriers at the organizations that will implement the intervention. In step 3 of the TRIP model, baseline performance on key metrics is measured and areas for improvement are identified based on those metrics. In step 4, an implementation intervention is launched to support the delivery of the evidence-based practice bundle. According to the TRIP model, the implementation intervention should be designed to overcome the implementation barriers identified in step 2 and address deficits in performance identified in step 3.

#### Pilot study design

This pilot study uses a single group pre/post-observational design. All study sites will use the CUSP implementation strategy to support the delivery of the guideline-concordant cardiovascular risk factor care processes included in the evidence bundle.

#### Setting and sample

Study sites will include five psychiatric rehabilitation programs implementing behavioral health homes in Maryland. Psychiatric rehabilitation programs are outpatient mental health programs that deliver psychosocial rehabilitation services such as vocational training and mental health case management. To qualify for psychiatric rehabilitation services in Maryland, individuals must be receiving care from a psychiatrist who determines that the client has significant functional impairment as a result of their mental illness.

In Maryland, psychiatric rehabilitation programs have historically been responsible for coordinating mental health, substance use, and social services, such as housing, employment, and nutrition support for their clients. In 2013, Maryland used the Affordable Care Act Medicaid Health Home waiver to implement behavioral health homes, adding physical health service coordination to the responsibilities of the psychiatric rehabilitation programs that chose to participate in the waiver program. In practice, this waiver allowed psychiatric rehabilitation programs to bill Medicaid on a per-client, per-month basis for physical health care management and coordination, which are not traditionally reimbursable services. At each site, behavioral health home implementation is led by a nurse care manager, with support from a medical director and consulting primary care physician or nurse practitioner. All psychiatric rehabilitation program clients covered by Medicaid are eligible to join the behavioral health home program. Enrollment is led by the nurse care manager at each site and clients actively consent to join the program. Our team’s prior research found that psychiatric rehabilitation programs work to enroll all eligible clients into their behavioral health home programs.

The present study will include five behavioral health home sites. Each site has approximately 30 staff, which includes all personnel—leaders, health home providers, psychiatric rehabilitation providers, and support staff, e.g., cafeteria workers—and up to 200 clients with SMI.

### Evidence-based practice bundle

Aligning with step 1 of the TRIP model, the study team conducted a comprehensive review of clinical guidelines and scholarly literature to create a “bundle” of evidence-based care processes for hypertension, dyslipidemia, and diabetes mellitus among people with SMI. As these conditions are highly co-morbid among people with SMI and involve cross-cutting care delivery processes, we developed a single bundle for all three conditions; the goal is for study sites to use CUSP to implement the evidence-based practices in the bundle. While clinical care guidelines are largely similar for cardiovascular risk factors in people with versus without SMI, some special considerations for this population (e.g., metabolic side effects of psychotropic medications) are needed. Further, care delivery processes often need to be tailored for people with SMI due to unique challenges faced by this group such as cognitive deficits and transportation barriers. The bundle includes two overarching components: clinical care processes and care coordination and management processes. The bundle developed by the study team was reviewed by four external clinical experts as well as two behavioral health home teams in Maryland and refined based on feedback. Examples of evidence-based practice bundle content are shown in Table 1. In the pilot study, a general internist with expertise in integrated care for SMI and an expert motivational interviewing trainer will deliver 8 h of training on the evidence bundle to site CUSP teams; motivational interviewing is an important component of the evidence bundle.

**Table 1:** Examples of evidence-based practice bundle content

CVD risk factor-specific care processes	Cross-cutting care processes
Hypertension: ensure repeat blood pressure check every 3 months if blood pressure is > 120/80	- Use database to track and prioritize screening, monitoring, and treatment of hypertension, diabetes, and dyslipidemia - Use primary care visit communication form to facilitate communication between primary care provider and behavioral health home team - Use motivational interviewing to engage consumers in their CVD risk factor care (e.g. resolving ambivalence around starting medication to treat blood pressure, self-management strategies for diabetes)
Dyslipidemia: obtain lipid panel every 12 months	
Diabetes mellitus: obtain HbA1c every 6 months if HbA1c < 7%	

### The Comprehensive Unit-Based Safety Program (CUSP) implementation strategy

The CUSP implementation strategy was developed by the Johns Hopkins University Armstrong Institute for Patient Safety and Quality (“Armstrong Institute”) as a strategy for improving inpatient safety [44]. CUSP was designed as a vehicle to implement the TRIP model for translating evidence into practice [49]. It includes provider training, expert facilitation, and implementation of a five-step quality improvement process. The five steps are as follows. First, all organization staff are trained on the science of quality improvement. Second, CUSP teams create a process at their organization for continuously identifying barriers to evidence-based care. Third, CUSP teams create and implement processes for engaging senior leaders in efforts to improve evidence-based practice delivery. Fourth, CUSP teams systematically address barriers to evidence-based care. Fifth, CUSP teams use CUSP tools to improve teamwork and communication. These steps are not necessarily linear and are often implemented in parallel with one another.

The CUSP strategy is designed to foster a team-based quality improvement culture where providers work together to identify a patient-centered problem (e.g., poor control of diabetes mellitus) and then to address barriers to receipt of evidence-based care for that problem [44, 45]. By training providers and putting standard processes to implement evidence-based care in place, CUSPs improve providers’ self-efficacy to deliver such care [44, 45]. Improvements in culture and self-efficacy should increase the delivery of evidence-based care and contribute to improved clinical outcomes [44, 45]. CUSPs have been shown to support significant improvements in inpatient safety and are endorsed by the US Agency for Healthcare Quality and Research (AHRQ) [46, 49–52]. The CUSP strategy is more recently being applied to outpatient quality improvement [53–56]. The study described in this protocol is the first to adapt CUSP to support the implementation of an integrated care model for people with SMI.

### Adapting CUSP for the behavioral health home context

CUSP was designed to improve inpatient safety, and the training materials and CUSP tools developed by the Armstrong Institute are oriented toward the inpatient context. The study team therefore adapted CUSP trainings and tools to enhance relevance to the community mental health organization context and to focus on physical health care coordination and management. The five core components of the CUSP model, described above, were not modified; rather, we adapted the training and toolkit materials to align with mental health organization culture, workflow, and resources and mental health provider roles, responsibilities, and skills. All adaptations were tracked using the FRAME model, a framework for delineating adaptations across multiple domains including whether the change was planned/proactive or unplanned/reactive, the type of adaptation, and the goal of the adaptation. Any additional adaptations made during implementation will also be tracked using this model [57].

For example, the original CUSP “Science of Safety training,” which is administered to all organization staff during CUSP rollout, was adapted to become the “Science of Quality Training.” The key concepts covered in the training (the historical and contemporary context of the science of patient safety/quality improvement; how system design affects system results; the principles of safe design and how they apply to technical work and teamwork; and the importance of diverse and independent input into quality-of-care efforts) were unchanged, but the motivating examples provided were changed from inpatient safety scenarios to examples of community mental health programs implementing physical health integration models. In another example, a core CUSP tool, the “learning from defects” tool—a worksheet that CUSP teams use to systematically identify the causes of and potential solutions to “defects” in care processes or systems that lead to adverse patient safety events—was adapted to become the “learning from challenges tool.” Instead of deconstructing an adverse patient safety event that occurred in the inpatient unit, the adapted tool is designed to help CUSP teams walk through a case in which a specific client did not receive optimal cardiovascular risk factor care, e.g., a case where a health home client with uncontrolled diabetes mellitus has not seen their primary care doctor in over a month.

### Piloting CUSP in behavioral health homes

CUSP is implemented by interdisciplinary CUSP teams comprising individuals from within the implementing organization. In this pilot study, sites will be encouraged to form teams that include a psychiatric rehabilitation program leader, a health home medical director, a nurse care manager leading behavioral health home implementation, an individual responsible for health IT and/or data management at the organization, and psychiatric rehabilitation program staff involved in behavioral health home implementation tasks such as driving clients to primary care appointments.

CUSP is implemented over three phases: pre-implementation, implementation, and sustainment. During the pre-implementation and implementation phases, sites receive support from an expert facilitator trained by the Armstrong Institute. In this pilot study, the pre-implementation phase will be 1–2 months, the implementation phase will be 12 months, and the sustainment phase will be 3 months at each study site.

The pre-implementation phase includes CUSP team training (Table 2) and TRIP model steps 2–3—identify implementation barriers and measure baseline performance. During this phase, the study team will lead focus groups with CUSP team members to identify initial implementation barriers and collect baseline measures for primary and secondary outcomes (details below). CUSP teams will then use focus group results and baseline data to inform quality improvement goals and to prioritize which elements of the evidence-based practice bundle that they plan to implement first. For example, if a site identifies a lack of a system for tracking diagnosis, monitoring, and treatment of cardiovascular risk factors among clients with SMI as a key barrier to care, they may prioritize implementation of a database to perform these functions; use of such a database to conduct population health management functions is one of the evidence-based processes included in the bundle.

**Table 2:** CUSP trainings

CUSP trainings	Who receives training	Estimated length
Science of quality training: introduces the principles of CUSP	All psychiatric rehabilitation program staff, including but not limited to CUSP team	1 h
CUSP Process Training-Advanced: module 1, introduction to CUSP; module 2, creating engagement and ownership of CUSP; module 3, identifying and learning from challenges; module 4, kicking off CUSP	CUSP team	Four 1-h sessions
Evidence bundle for cardiovascular risk factors and care coordination/management	CUSP team	Two 2-h sessions
Evidence bundle motivational interviewing for CVD risk factor management	CUSP team	Two 2-h sessions
Sustaining a CUSP team	CUSP team	60 min

In the implementation phase, sites implement the five-step CUSP process with support, through monthly meetings and ad hoc requests for assistance, from the CUSP expert facilitator. CUSP teams and the expert facilitator will work together to conduct the introductory science of quality improvement training for all psychiatric rehabilitation program staff (step 1). To implement a process for collecting input from all psychiatric rehabilitation program staff on barriers to delivery of evidence-based cardiovascular care (step 2), the CUSP team will administer the *Staff Input* CUSP tool at an all-hands meeting every 6 months, or more frequently as needed. This CUSP worksheet asks staff to identify the ways in which their role at the organization might influence clients’ cardiovascular care and health (e.g., assisting with medical appointment transportation, providing medication monitoring, offering healthier food options in on-site meals) and to identify challenges they see in helping clients prevent and/or manage cardiovascular risk factors. The CUSP team uses this input to inform their efforts to overcome challenges and implement the evidence bundle.

In the inpatient setting, hospital administrators are often not CUSP team members. Thus, other strategies are used to engage senior leaders (step 3), such as “walk-rounds” where senior administrative leaders walk through the care delivery process with front-line providers to understand systemic issues—which often require facility updates, staffing changes, or additional resources, all of which are the purview of senior leadership—contributing to adverse patient safety events. In contrast to hospitals with large numbers of leaders and staff, psychiatric rehabilitation programs in Maryland typically have about 30 total employees. Thus, psychiatric rehabilitation program directors will directly engage by serving as members of the CUSP team.

The fourth CUSP step, “systematically address barriers to evidence-based care,” is where the core CUSP work occurs. CUSP teams will conduct monthly meetings in which they use the *Learning from Challenges* tool referenced previously to review a case in which a client did not receive the guideline-concordant cardiovascular risk factor care processes included in the evidence bundle, identify the challenges that contributed to the suboptimal care, develop solutions to overcome those challenges, and create a plan for beginning to implement those solutions over the coming month. Critically, the goal is not just to address the problem experienced by an individual client but to enhance systems to prevent the issue from happening to all clients in the future. In other words, if client X was hospitalized with uncontrolled diabetes and the CUSP team learns that she missed three primary care appointments in the 2 months prior to the hospitalization, the goal is not only to make sure client X receives needed outpatient care but also to set up a system to make sure all clients with poorly controlled diabetes have regularly scheduled primary care visits and that clients are supported in attending those visits through reminders, transportation, accompaniment to the appointment, etc. Step 5 of CUSP, use CUSP tools to improve teamwork and communication, is ongoing across CUSP phases as noted with the *Staff Input* and *Learning from Challenges* tools above (see Table 3 for additional CUSP tools).

**Table 3:** CUSP tools

CUSP tools	Tool purpose	CUSP phase
Roles and Responsibilities Tool	Helps implementing organizations identify interdisciplinary CUSP team members	Pre-implementation
Pre-Mortem Tool	Facilitates the development of proactive strategies to overcome likely implementation challenges by helping CUSP teams imagine why implementation of the evidence-based practice bundle might fail at their organization	Pre-implementation
Staff Input Tool	Helps staff identify the ways in which their role at the organization might influence the delivery of the evidence-based practice bundle	Implementation and sustainment
Learning from Challenges Tool	Supports identification of causes of and solutions to challenges in implementing the evidence-based practice bundle	Implementation and sustainment
Monthly Meeting Agenda Tool	Provides a template for developing monthly CUSP meeting agendas	Implementation and sustainment
Medical Debrief Tool	Provides a template for weekly CUSP team reviews of service needs among clients with poorly controlled cardiovascular risk factors	Implementation and sustainment

In the sustainment phase, CUSP teams continue implementing CUSP without support from the expert facilitator. While expert facilitation is discontinued in the sustainment phase, CUSP teams have ongoing access to the Armstrong Institute’s CUSP network and resources. Eventually, the goal is for CUSP teams to use the CUSP process to implement additional evidence-based practices. After using CUSP to implement the cardiovascular risk factor evidence bundle in the present study, teams could use CUSP to support the implementation of other evidence-based practices, for example, delivery of smoking cessation treatment.

### Data collection

Data collection will include focus groups, surveys, and client health record abstraction (see Table 4 for a summary of data collection and measures). Focus groups with CUSP team members will be led by a study team member trained in qualitative data collection at baseline and 15-month follow-up, the end of the sustainment phase. Focus groups will be conducted using a semi-structured guide designed to elicit perceptions of barriers and facilitators to implementation of CUSP and the evidence-based cardiovascular risk factor care bundle. Focus groups will include approximately five members, be approximately 60 min in length and be audio-recorded and transcribed. Surveys will be administered via email with instructions and a link to the survey in the REDCap software or by paper-and-pencil, depending upon site preferences. Two groups of providers will be surveyed: (1) all psychiatric rehabilitation program staff (approximately 30 per site) will complete a survey measuring quality improvement culture and (2) CUSP team members (approximately 10 per site) will complete the survey measuring self-efficacy, appropriateness, acceptability, and feasibility; below are the descriptions of these measures. Client health record abstraction will be conducted by the CUSP team. The CUSP team will abstract data from existing systems (e.g., electronic health records, paper files, other psychiatric rehabilitation program client tracking systems) to complete a spreadsheet with all client health and demographic measures (described below) created by the study team. Data will be abstracted for all behavioral health home clients (approximately 200 per site).

**Table 4:** Primary and secondary outcome measures

Measures	Participants	Timing
**Qualitative data collection**: conducted by a study team member trained in qualitative data collection.		
**CUSP team focus groups**	Selected CUSP team members (~ 5 per site)	Baseline, 15 months
**Survey measures**: collected through surveys administered by the study team		
**Quality improvement culture:** measured using a modified version of the validated Survey on Patient safety used in inpatient CUSPs	All psychiatric rehabilitation program staff (~ 30 per site)	Baseline, 12 months
**Self-efficacy to deliver evidence-based CVD risk factor care coordination and management**: measured using a modified version of Compeau and Higgins’ task-focused self-efficacy scale	CUSP team (~ 10 per site)	Baseline, 12 months
**Acceptability, appropriateness, and feasibility of the intervention implementation strategies**: measured with a brief 4-item practice instrument (AIM, IAM, FIM)	CUSP team (~ 10 per site)	Baseline, 12 months
**Acceptability, appropriateness, and feasibility of the evidence-based practice bundle:** measured with a brief 4-item practice instrument (AIM, IAM, FIM)	CUSP team (~ 10 per site)	Baseline, 12 months
**Demographic characteristics:** age, sex, race, ethnicity, length of time at the program, role in the program, previous motivational interviewing training, comfort with technology	All psychiatric rehabilitation program staff (~ 30 per site)	Baseline
**Health home client measures**: collected by CUSP team/ health home staff during regular health home program care		
**CVD risk factors:** weight, BMI, tobacco smoking status, blood pressure, diabetes, HgbA1c, lipids, diagnoses of hypertension, diabetes, dyslipidemia, CVD risk scores	All behavioral health home participants at the psychiatric rehabilitation program (~ 200 per site)	Baseline and 6 and 12 months
**Evidence-based CVD risk factor care**: % of consumers eligible to receive care processes in the evidence bundle who receive that process (e.g., diabetic eye or foot exam)		
**CVD risk factor control**: % of consumers meeting clinical criteria for having controlled hypertension dyslipidemia, diabetes		
**Demographic and clinical characteristics**: age, gender, race, ethnicity, living arrangement, disability status, transportation issues, mental health diagnoses, medications used		

### Measures

#### Primary outcomes

Primary outcome measures are organization quality improvement culture and provider self-efficacy to deliver the evidence-based practices in the bundle, the mechanisms by which CUSP has been shown in prior work to influence care quality and client health outcomes. We will measure the change in these outcomes from baseline to 12 months.

Quality improvement culture will be measured using an adapted version of the validated Survey on Patient Safety, which was designed to measure safety culture in inpatient units. Our study team adapted the survey items to pertain to community mental health settings. The 34-item survey includes measures in 9 domains: teamwork, supervisor promotion of quality improvement, organizational learning, management support for quality improvement, feedback and communication, communication openness, mistake reporting, staffing capacity, and overall perceptions of quality improvement culture. Each of the items includes response options on a 5-point Likert scale. An average quality improvement culture score is calculated by summing responses across all items and dividing by the total number of items. A higher average scores signifies organizational culture more conducive to quality improvement. This survey measure will be completed by all psychiatric rehabilitation program staff.

Self-efficacy will be measured using an adapted version of Compeau and Higgins’ task-focused self-efficacy scale. Providers will complete three separate versions of the nine-item self-efficacy instrument, one assessing self-efficacy to coordinate guideline-concordant care for clients with SMI with hypertension, one for dyslipidemia, and one for diabetes mellitus. Each of the nine items includes response options on a 10-point Likert scale. An average score is calculated by summing responses across all nine items and dividing by the total number of items. A higher score signifies greater self-efficacy. This survey measure will be completed by all CUSP team members.

#### Secondary outcomes

Secondary outcomes include implementers’ perceptions of the acceptability, appropriateness, and feasibility of the CUSP implementation strategy; client receipt of evidence-based care for hypertension, dyslipidemia, and diabetes mellitus; and client hypertension, dyslipidemia, and diabetes mellitus control. We will measure the change in these outcomes from baseline to 6 and 12 months.

Acceptability, appropriateness, and feasibility will be measured using three validated survey measures: the Acceptability of Intervention Measure (AIM), Intervention Appropriateness Measure (IAM), and Feasibility of Intervention Measure (FIM). Each of these measures includes four items measured on a 5-point Likert scale. An average score for each measure is calculated by summing responses across all four items and dividing by the total number of items. A higher score signifies greater acceptability, appropriateness, or feasibility. These survey measures will be completed by all CUSP team members.

Measures of client receipt of evidence-based cardiovascular risk factor care will include the percent of eligible consumers who receive each of the following evidence-based care practices: percentage of clients with SMI with poorly controlled hypertension (see definition below) who had a follow-up blood pressure measurement within 3 months; percentage of clients with SMI diagnosed with diabetes mellitus who received each of the following in the past 12 months: a lipid panel, statin therapy, a dilated eye exam, a foot exam, and a urine-protein-creatinine test; percentage of clients with SMI diagnosed with diabetes mellitus who had a HbA1c measurement in the past 6 months; and percentage of clients with SMI diagnosed with dyslipidemia who received a lipid panel in the past 12 months. We will also measure the percentage of clients with SMI diagnosed with each of the three cardiovascular risk factors of interest who received diet and exercise counseling.

Measures of cardiovascular risk factor control will include the percentage of clients with SMI diagnosed with hypertension who have controlled hypertension, defined as blood pressure < 130/80 mmHg; percentage of clients with SMI diagnosed with dyslipidemia who have controlled dyslipidemia, defined as low-density lipoprotein total cholesterol < 200 mg/dl and LDL < 130 mg/dl (primary measure) or on a statin (secondary measure); and the percentage of clients with SMI diagnosed with diabetes mellitus with controlled diabetes, defined as HbA1c < 7.0.

#### Fidelity

Fidelity of implementation of CUSP to support the delivery of the cardiovascular risk factor evidence bundle will be measured using the Stages of Implemented Completion (SIC) tool [58]. The SIC assesses milestone achievement across eight stages of implementation; these stages have been shown to be generalizable across multiple types of interventions. We tailored the universal SIC measure to include specific milestones relevant to the proposed study. The SIC will be updated on a weekly basis by the study coordinator, who will track the dates of completion of each milestone at each study site.

#### Implementation barriers and facilitators

Measures of CUSP team members’ perceptions of implementation barriers and facilitators will be coded in the focus group data. Baseline measures will focus on pre-implementation perceptions of barriers and facilitators. Fifteen-month measures will focus on perceptions of barriers and facilitators following the expert-facilitated implementation phase and the 3-month sustainment phase without expert facilitation.

#### Provider and client characteristics

We will use survey measures to collect information on psychiatric rehabilitation program providers’ age, gender, race, ethnicity, length of time working at the program, and role in the program. We will use client record abstraction to collect data on clients’ diagnoses of hypertension, dyslipidemia, and diabetes mellitus as well as their mental health and substance use disorder diagnoses; tobacco use; height and weight; medications prescribed; and sociodemographic characteristics including age, gender, race, ethnicity, living arrangement, disability status, and transportation needs.

### Analysis

#### Qualitative data analysis

After each focus group, the study team member leading the group will create a summary memo. This summary memo will help to identify preliminary themes within the data. Focus group transcripts will then be analyzed using a staged approach, which begins with general coding and then evolves to include more specific codes. The study team will create an initial codebook based on the research questions and focus group summary memos. Two coders will pilot the codebook using a random sub-sample of transcripts. Additional themes that emerge during the pilot phase will be added to the codebook. Disagreement between the two coders will be resolved through a discussion with the full study team. The finalized codebook will be applied to all focus group transcripts, and qualitative research software will be used to organize text segments according to themes and sub-themes. We will conduct member-checking by asking all focus group participants to provide feedback on a memo summarizing key themes.

#### Quantitative data analysis

We will conduct two main analyses. First, we will compare pre/post (baseline, 12 months) quality improvement culture and provider self-efficacy, the mechanisms through which CUSP is designed to improve the delivery of evidence-based care. Second, we will examine the changes in clients’ receipt of evidence-based care for hypertension, dyslipidemia, and diabetes mellitus and cardiovascular risk factor control from baseline to 6 and 12 months after implementing CUSP, controlling for client characteristics. For both analyses, we will use linear mixed effects modeling approaches for continuous outcomes and generalized estimated equations for binary outcomes. The models will include pre/post-CUSP indicators, fixed effects for the five study sites, and provider/client characteristics. We will use an unstructured variance-covariance model allowing different variances at outcome measurements over time. In addition to these main analyses, we will examine CUSP team perceptions of intervention acceptability, appropriateness, and feasibility descriptively at baseline and at 12-month follow-up.

#### Addressing potential bias

For qualitative analyses, two coders will independently code a random sub-sample of transcripts to generate the initial codebook and reconcile disagreements with the full study team. The member-checking described above will also help avoid bias in the study team’s interpretation of the data. For quantitative data, social desirability is a threat to survey measures of quality improvement culture and self-efficacy. To minimize this threat, survey responses will be confidential and reported only in aggregate form, processes that will be noted in the informed consent process. Measurement error is a threat to analyses of client record data given that sites have varying levels of data reporting infrastructure. We work to minimize this threat by having all sites report data in a standard database developed by the study team. This database will be quality-checked throughout the study by research staff.

## Discussion

Recent financing mechanisms to support integrated mental-physical healthcare models, such as the Affordable Care Act Medicaid Health Home waiver used to finance the Maryland behavioral health home programs participating in this pilot study, create a critical infrastructure to support the adoption of care integrated models. This pilot study tests the potential for the CUSP implementation strategy, which has been shown to be effective and sustainable in the inpatient safety context, to support evidence-based care coordination and management for hypertension, dyslipidemia, and diabetes mellitus among clients with SMI participating in behavioral health home programs.

Qualitative and survey research suggests that poor alignment between specialty mental health program culture, which has historically focused strictly on mental health treatment, and behavioral health homes’ physical-health oriented goals is a threat to successful behavioral health home implementation. The lack of structured protocols for conducting effective physical health care coordination and management is another key implementation barrier; behavioral health homes in Maryland and other states do not currently use this type of standard protocol, resulting in a high degree of variation across sites. Standard protocols have the potential to promote self-efficacy to conduct physical health coordination and management among behavioral health home staff, many of whom do not have prior training or experience in these domains. In the inpatient safety context, the CUSP implementation strategy has been shown to support cultural shifts and implementation of standard processes and systems for the delivery of evidence-based services.

### Strengths and limitations

While CUSP is an intensive implementation strategy, behavioral health home teams already meet regularly and work together to improve care; the CUSP strategy is designed to help these teams work more efficiently and effectively. Our pilot study is designed to collect in-depth implementation data. Study sites are committed to participation and will be compensated for their time and effort for focus groups and surveys. While CUSP directly targets key cultural and process barriers to physical health care coordination and management in behavioral health home programs, it does not directly address other important barriers including inadequate financing and poorly integrated specialty mental health and general medical health IT systems. The inclusion of organization leadership in CUSP teams is designed to support strategies to overcome these significant system-level barriers, but progress on these dimensions will likely be long-term. Our single group, pre/post-pilot study design does not allow us to make causal inferences about the effects of the CUSP implementation strategy on outcomes. This pilot study is designed to inform the development of a future randomized clinical trial.

## Conclusion

Team-based care is increasingly used in integrated mental-physical healthcare models across the country, making the CUSP implementation strategy relevant for a wide variety of settings. While we apply CUSP to the implementation of evidence-based hypertension, dyslipidemia, and diabetes care, our evidence bundle can be updated in the future to include other issues such as smoking cessation treatment. CUSP is designed to be fully integrated into organizations, sustained indefinitely, and used to continually improve evidence-based practice delivery.

## Data Availability

Please contact the authors for data requests.

## Abbreviation

CUSP: Comprehensive Unit-Based Safety Program
